# Supplementary material for: Polyketide synthase gene clusters in the ericoid mycorrhizal fungus Oidiodendron maius: genome-wide prediction, expression profiling, and functional characterization of knockout mutants
Source: Front Plant Sci. 2026 Sep 16;17:1924430. doi: 10.3389/fpls.2026.1924430 (PMC13623903; doi:10.3389/fpls.2026.1924430)
Supplement: Supplementary file 1 [file SupplementaryFile1.zip › Supplementary Materials/Supplementary Folder 1.zip!/Oidma1_AssemblyScaffolds_Repeatmasked/knownclusterblast/cluster51/jgi.p_Oidma1_35767_mibig_hits.html]

| MIBiG Protein | Description | MIBiG Cluster | MiBiG Product | % ID | % Coverage | BLAST Score | E-value |
| --- | --- | --- | --- | --- | --- | --- | --- |
| ADI24937.1 | VrtK | BGC0000168 | Viridicatumtoxin\_biosynthetic\_gene\_cluster | 58 | 93.7131630648 | 599 | 2.8e-171 |
| AAC49200.1 | putative\_p450\_monooxygenase | BGC0000152 | Sterigmatocystin\_biosynthetic\_gene\_cluster | 39 | 93.9096267191 | 371 | 1.2e-102 |
| AAM18769.1 | AflL | BGC0000011 | Aflatoxin/sterigmatocystin\_biosynthetic\_gene\_cluster | 39 | 97.2495088409 | 368 | 1e-101 |
| AAS90059.1 | VerB | BGC0000009 | Aflatoxin\_biosynthetic\_gene\_cluster | 39 | 94.8919449902 | 366 | 4e-101 |
| AAS90011.1 | VerB | BGC0000007 | Aflatoxin\_biosynthetic\_gene\_cluster | 39 | 95.0884086444 | 364 | 1.5e-100 |
| AAS90102.1 | VerB | BGC0000006 | Aflatoxin\_biosynthetic\_gene\_cluster | 39 | 95.8742632613 | 363 | 5.7e-100 |
| AAS90079.1 | VerB | BGC0000010 | Aflatoxin\_biosynthetic\_gene\_cluster | 39 | 95.8742632613 | 363 | 5.7e-100 |
| BAE71326.1 | desaturase | BGC0000004 | Aflatoxin\_biosynthetic\_gene\_cluster | 38 | 95.0884086444 | 363 | 5.7e-100 |
| AAS90033.1 | VerB | BGC0000008 | Aflatoxin\_biosynthetic\_gene\_cluster | 39 | 94.1060903733 | 361 | 1.3e-99 |
| AAS90010.1 | AvnA | BGC0000007 | Aflatoxin\_biosynthetic\_gene\_cluster | 38 | 92.9273084479 | 322 | 1.1e-87 |
| AAS90078.1 | AvnA | BGC0000010 | Aflatoxin\_biosynthetic\_gene\_cluster | 38 | 92.9273084479 | 320 | 3.2e-87 |
| AAS90101.1 | AvnA | BGC0000006 | Aflatoxin\_biosynthetic\_gene\_cluster | 38 | 92.9273084479 | 319 | 9.5e-87 |
| AAS90032.1 | AvnA | BGC0000008 | Aflatoxin\_biosynthetic\_gene\_cluster | 38 | 92.9273084479 | 318 | 1.6e-86 |
| BAE71325.1 | cytochrome\_P450\_monooxygenase | BGC0000004 | Aflatoxin\_biosynthetic\_gene\_cluster | 37 | 92.9273084479 | 315 | 1.4e-85 |
| AAS90058.1 | AvnA | BGC0000009 | Aflatoxin\_biosynthetic\_gene\_cluster | 37 | 94.4990176817 | 306 | 6.3e-83 |
| ACH72905.1 | AflG | BGC0000011 | Aflatoxin/sterigmatocystin\_biosynthetic\_gene\_cluster | 36 | 94.1060903733 | 297 | 2.9e-80 |
| AAC49196.1 | putative\_p450\_monooxygenase | BGC0000152 | Sterigmatocystin\_biosynthetic\_gene\_cluster | 36 | 90.9626719057 | 294 | 2.5e-79 |
| AAK33080.1 | putative\_trichothecene\_C-15\_hydroxylase | BGC0001277 | Nivalenol\_/\_deoxynivalenol\_/\_3-acetyldeoxynivalenol\_/ | 34 | 97.4459724951 | 290 | 4.7e-78 |
| AAK33070.1 | trichothecene\_C-15\_hydroxylase | BGC0001278 | Nivalenol\_/\_deoxynivalenol\_/\_3-acetyldeoxynivalenol\_/ | 34 | 97.6424361493 | 289 | 1e-77 |
| AAK53587.1 | isotrichodermin\_C-15\_hydroxylase | BGC0000931 | Trichothecene\_biosynthetic\_gene\_cluster | 34 | 97.4459724951 | 288 | 1.8e-77 |
| AAN74815.2 | Fum2p | BGC0000062 | Fumonisin\_biosynthetic\_gene\_cluster | 34 | 97.6424361493 | 286 | 5.2e-77 |
| AAK53580.1 | isotrichodermin\_C-15\_hydroxylase | BGC0000930 | Trichothecene\_biosynthetic\_gene\_cluster | 34 | 97.4459724951 | 285 | 1.5e-76 |
| ACD39773.1 | cytochrome\_P450 | BGC0000134 | Radicicol\_biosynthetic\_gene\_cluster | 33 | 97.6424361493 | 280 | 2.9e-75 |
| Bcin12g06370.1 |  | BGC0000631 | Botrydial\_biosynthetic\_gene\_cluster | 37 | 93.3202357564 | 279 | 8.3e-75 |
| OAQ83755.1 | isotrichodermin\_C-15\_hydroxylase | BGC0001358 | Leucinostatins\_biosynthetic\_gene\_cluster | 34 | 99.8035363458 | 278 | 1.8e-74 |
| ACD39760.1 | cytochrome\_P450 | BGC0000077 | Hypothemycin\_biosynthetic\_gene\_cluster | 35 | 94.695481336 | 273 | 4.6e-73 |
| ACD39751.1 | cytochrome\_P450 | BGC0000076 | Hypothemycin\_biosynthetic\_gene\_cluster | 35 | 94.695481336 | 273 | 4.6e-73 |
| EFY94501.1 | benzoate\_4-monooxygenase\_cytochrome\_P450 | BGC0000337 | Destruxins\_biosynthetic\_gene\_cluster | 33 | 96.8565815324 | 272 | 1.3e-72 |
| BAD29972.1 | P450\_monooxygenase\_1 | BGC0000676 | Aphidicolin\_biosynthetic\_gene\_cluster | 33 | 97.6424361493 | 260 | 5.2e-69 |
| XP\_002384857.1 | cytochrome\_P450,\_putative | BGC0001234\_c2 | 4,4'-piperazine-2,5-diyldimethyl-bis-phenol\_biosynthetic\_gene | 32 | 95.8742632613 | 253 | 6.4e-67 |
| BAD83680.1 | cytochrome\_P-450 | BGC0000012 | Alternapyrone\_biosynthetic\_gene\_cluster | 31 | 98.6247544204 | 250 | 5.4e-66 |
| ACZ66254.1 | APS8 | BGC0000304 | Apicidin\_biosynthetic\_gene\_cluster | 26 | 88.605108055 | 165 | 1.7e-40 |
| OAQ83757.1 | cytochrome\_P450 | BGC0001358 | Leucinostatins\_biosynthetic\_gene\_cluster | 26 | 91.1591355599 | 156 | 6.2e-38 |
| AAZ95016.1 | cytochrome\_P450\_monooxygenase | BGC0000048 | Dothistromin\_biosynthetic\_gene\_cluster | 26 | 93.3202357564 | 145 | 1.9e-34 |
| ctg1\_orf5 |  | BGC0000687 | Phyllocladan-16alpha-ol\_biosynthetic\_gene\_cluster | 34 | 28.8801571709 | 82 | 1.9e-15 |
| CBW45722.1 | putative\_cytochrome\_P450\_hydroxylase | BGC0000952\_c3 | Pristinamycin\_biosynthetic\_gene\_cluster | 25 | 71.1198428291 | 52 | 2.2e-06 |
